# Differential Functional Roles of ALDH1A1 and ALDH1A3 in Mediating Metastatic Behavior and Therapy Resistance of Human Breast Cancer Cells

**DOI:** 10.3390/ijms18102039

**Published:** 2017-09-22

**Authors:** Alysha K. Croker, Mauricio Rodriguez-Torres, Ying Xia, Siddika Pardhan, Hon Sing Leong, John D. Lewis, Alison L. Allan

**Affiliations:** 1 London Regional Cancer Program, London Health Sciences Centre, 790 Commissioners Road East, London, ON N6A 4L6, Canada; alysha.croker@gmail.com (A.K.C.); rodrimauricio@gmail.com (M.R.-T.); ying.xia@lhsc.on.ca (Y.X.); 2 Department of Anatomy & Cell Biology, Schulich School of Medicine & Dentistry, Western University, London, ON N6A 5C1, Canada; 3 Department of Surgery, Schulich School of Medicine & Dentistry, Western University, London, ON N6A 5C1, Canada; siddika15@gmail.com (S.P.); honsing.leong@gmail.com (H.S.L.); 4 Department of Oncology, University of Alberta, 5-142C Katz Group Building, 114th St. and 87th Ave. S., Edmonton, AB T6G 2E1, Canada; jdlewis@ualberta.ca; 5 Department of Oncology and Anatomy & Cell Biology, Schulich School of Medicine & Dentistry, Western University, London, ON N6A 5C1, Canada; 6 Cancer Research Laboratory Program, Lawson Health Research Institute, 750 Base Line Road, Suite 300, London, ON N6C 2R5, Canada

**Keywords:** breast cancer, metastasis, therapy resistance, ALDH1A1, ALDH1A3

## Abstract

Previous studies indicate that breast cancer cells with high aldehyde dehydrogenase (ALDH) activity and CD44 expression (ALDH^hi^CD44^+^) contribute to metastasis and therapy resistance, and that ALDH1 correlates with poor outcome in breast cancer patients. The current study hypothesized that ALDH1 functionally contributes to breast cancer metastatic behavior and therapy resistance. Expression of ALDH1A1 or ALDH1A3 was knocked down in MDA-MB-468 and SUM159 human breast cancer cells using siRNA. Resulting impacts on ALDH activity (Aldefluor^®^ assay); metastatic behavior and therapy response in vitro (proliferation/adhesion/migration/colony formation/chemotherapy and radiation) and extravasation/metastasis in vivo (chick choroiallantoic membrane assay) was assessed. Knockdown of ALDH1A3 but not ALDH1A1 in breast cancer cells decreased ALDH activity, and knockdown of ALDH1A1 reduced breast cancer cell metastatic behavior and therapy resistance relative to control (*p* < 0.05). In contrast, knockdown of ALDH1A3 did not alter proliferation, extravasation, or therapy resistance, but increased adhesion/migration and decreased colony formation/metastasis relative to control (*p* < 0.05). This is the first study to systematically examine the function of ALDH1 isozymes in individual breast cancer cell behaviors that contribute to metastasis. Our novel results indicate that ALDH1 mediates breast cancer metastatic behavior and therapy resistance, and that different enzyme isoforms within the ALDH1 family differentially impact these cell behaviors.

## 1. Introduction

Breast cancer is a leading cause of death in women, due primarily to ineffective treatment of metastatic disease. In order to reduce mortality from breast cancer, it is therefore essential to learn more about the metastatic process, and in particular, mechanisms that may contribute to therapy resistance and disease progression [1,2].

Metastasis is a complex process that involves tumor dissemination from the primary tumor to distant sites throughout the body, arrest and extravasation at secondary organ sites, and initiation and maintenance of growth of metastatic lesions [1,3,4]. Given the multi-step nature of this process, it is not surprising that metastasis is highly inefficient, with the main rate-limiting steps being initiation of growth at the secondary site from single tumor cells to micrometastases, and maintenance of that growth into clinically detectable macrometastases [1,3,4,5]. Given the heterogeneous nature of breast cancer, this metastatic inefficiency suggests that only a small subpopulation of tumor cells can successfully navigate the entire metastatic process to successfully form metastases. We have previously identified such a subset of breast cancer cells with high aldehyde dehydrogenase (ALDH) activity and expression of CD44, and demonstrated that these ALDH^hi^CD44^+^ cells have enhanced tumor-initiating and metastatic abilities both in vitro and in vivo [6]. Subsequent studies by Charafe-Jauffret et al. (2009, 2010) supported our findings, indicating that ALDH^hi^CD44^+^ cells may have a role as metastasis-initiating cells [7,8]. We have also demonstrated that these ALDH^hi^CD44^+^ cells are significantly more resistant to chemotherapy and radiation therapy, and that the observed therapy resistance may occur, at least in part, via ALDH-dependent mechanisms [9].

The ALDH superfamily of enzymes is involved in detoxification and/or bioactivation of various intracellular aldehydes in a NAD(P)^+^-dependent manner [10,11]. Of particular biological importance, the ALDH1 family of enzymes (namely ALDH1A1 and ALDH1A3) plays an important role in oxidizing vitamin A (retinal) to retinoic acid (RA) through an alcohol intermediary. RA functions as a ligand for nuclear retinoid receptors and leads to transactivation and transrepression of target genes, and is finally degraded by CYP26 enzymes [12]. ALDH activity has been shown to be involved in self-protection of normal stem cells and in resistance to the chemotherapeutic drug cyclophosphamide [13]. In the treatment of acute promyelocytic leukemia (APL), the differentiation agent all-*trans* retinoic acid (ATRA) is used clinically in combination with chemotherapy [14,15]. Increased levels of RA signaling from ATRA treatment have been shown to indirectly suppress *ALDH1* promoter activity in liver cells [16], as well as driving the differentiation of promyelocytes into neutrophils, causing enhanced cell-cycle arrest and apoptosis [17]. Additionally, ATRA has been shown to modulate cell growth, apoptosis, and differentiation of breast cancer cells [18]. In terms of therapy resistance, Tanei et al. (2009) conducted a clinical study looking at 108 breast cancer patients who received neoadjuvant paclitaxel and epirubicin-based chemotherapy [19]. When ALDH1A1^+^ and CD24^−^CD44^+^ expression was compared between core needle biopsies (pre-treatment) and subsequent excision (post-treatment), there was a significant increase in ALDH1A1 positive cells, but no change in CD24^−^CD44^+^ cells, indicating that ALDH1A1^+^ cells may play a significant role in resistance to chemotherapy.

High ALDH1 expression has been shown to correlate with poor prognosis in breast cancer patients [20], and has been associated with early relapse, metastasis development, therapy resistance and poor clinical outcome [7,8,21,22,23]. The ALDH1A1 isozyme has been shown to have increased expression in breast cancer patients who present with positive lymph nodes and in patients who succumb to their disease [24]. In a meta-analysis that looked at almost 900 breast cancer cases compared to over 1800 control samples, Zhou et al. (2010) found that ALDH1A1 expression was significantly associated with a high histological grade, ER/PR negativity, HER2 positivity, and worse overall survival [25]. Furthermore, when ALDH^bright^ cells in various tumors, including breast, are treated with ALDH1A1-specific CD8^+^ T cells which target and eliminate ALDH1A1-positive cells, inhibition of tumorigenic and metastatic growth is observed [26]. In contrast, Marcato et al. (2011) demonstrated that ALDH1A3 (but not ALDH1A1) expression in patient breast tumors correlates significantly with tumor grade, metastasis, and cancer stage, indicating that even within the ALDH1 family, alternate isozymes may function differently [27]. Thus, in addition to the classical role of ALDH as a detoxification enzyme, growing evidence suggests that it may also be playing an additional role in disease progression.

The goal of the current study was to test the hypothesis that ALDH1 is not simply a marker of highly aggressive breast cancer cells and poor patient prognosis, but that it also contributes functionally to metastatic behavior and therapy resistance. Importantly, we wanted to begin to elucidate the differential roles of ALDH1 isozymes, namely ALDH1A1 and ALDH1A3. The novel findings presented here indicate that ALDH1 is functionally involved in breast cancer metastasis and therapy resistance, and that different isozymes within the ALDH1 family differentially impact these cell behaviors.

## 2. Results

### 2.1. Treatment with DEAB (Diethylaminobenzaldehyde) Reduces Breast Cancer Cell Proliferation, Adhesion, Migration, and Colony Formation In Vitro

**Figure 1:**
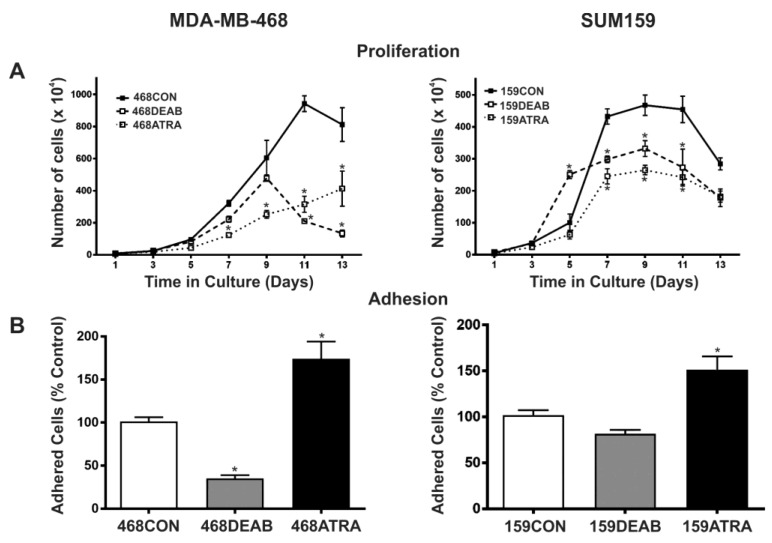

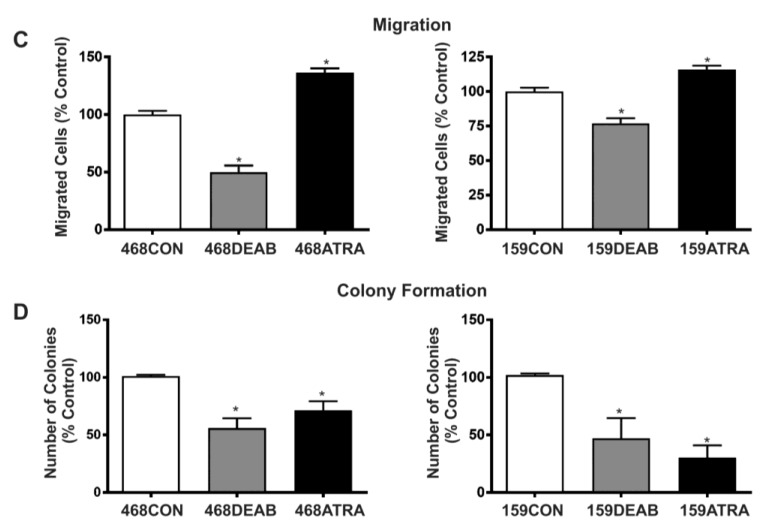
Treatment with diethylaminobenzaldehyde (DEAB) reduces breast cancer cell (**A**) proliferation, (**B**) adhesion, (**C**) migration, and (**D**) colony formation in vitro. MDA-MB-468 (left panels) and SUM159 (right panels) human breast cancer cells were treated with 5 μM all-*trans* retinoic acid (ATRA), 100 μM DEAB or ethanol (EtOH) as a vehicle control (CON). In all cases, data represents the mean ± standard error of the mean (SEM) normalized to vehicle control. * = significantly different than respective vehicle control treatment (*p* < 0.05).

We first investigated whether treating cells with previously established chemical inhibitors of ALDH would have a functional effect on malignant breast cancer cell behavior in vitro, including proliferation, adhesion, migration, and colony formation. This included treatment with a direct competitive substrate of ALDH (diethylaminobenzaldehyde (DEAB)) [28]), as well as the differentiation agent ATRA which has been shown to reduce ALDH promoter activity [9,16]. We observed that cells treated with either ATRA or DEAB demonstrated decreased growth in normal culture relative to respective vehicle control (EtOH) treated cells (*p* < 0.05) (Figure 1A). MDA-MB-468 cells treated with DEAB were significantly less adherent (Figure 1A) and migratory (Figure 1C) than vehicle control cells, and DEAB-treated SUM159 cells also demonstrated a significant decrease in migration (*p* < 0.05) (Figure 1C). In contrast, MDA-MB-468 and SUM159 cells treated with ATRA were observed to be significantly more adherent (*p* < 0.01) (Figure 1B) and migratory (Figure 1C) than respective control cells (*p* < 0.05). Finally, in keeping with the proliferation results, cells treated with either ATRA or DEAB demonstrated decreased colony formation in soft agar relative to vehicle control cells (*p* < 0.05) (Figure 1D).

### 2.2. Decreased Expression of ALDH1A3 but Not ALDH1A1 Reduces ALDH Activity as Measured by the ALDEFLUOR^®^ Assay

Rather than being direct inhibitors of ALDH isozyme expression, DEAB is a competitive substrate of ALDH [28] and ATRA inhibits ALDH promoter activity indirectly through the retinoic acid pathway. In support of this, we did not observe any significant effect of these inhibitors on directly reducing ALDH1A1 or ALDH1A3 protein expression (Figure S1). However, given that previous studies have demonstrated that expression of ALDH1A1 versus ALDH1A3 isozymes have differential correlation with tumor grade, metastasis, and cancer stage in breast cancer patients [27], we wanted to test the hypothesis that directly inhibiting ALDH using the alternative approach of targeted knockdown of ALDH1A1 or ALDH1A3 would also reduce proliferation, adhesion, migration, and colony formation of breast cancer cells.

**Figure 2:**
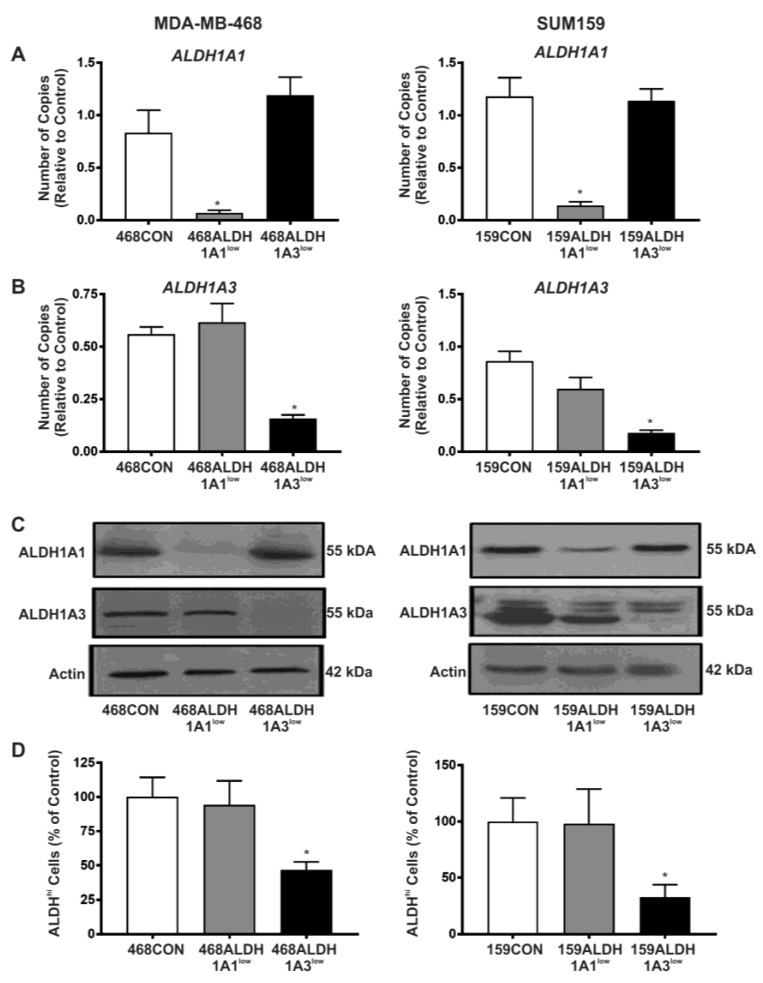
Decreased expression of ALDH1A3 but not ALDH1A1 reduces ALDH activity as measured by the Aldefluor^®^ assay. MDA-MB-468 (left panels) or SUM159 (right panels) human breast cancer cells were transfected with 100 pmol siRNA pool targeted towards ALDH1A1, ALDH1A3, or a scrambled control using Lipofectamine to generate the following cell lines: 468CON, 468ALDH1A1^low^, 468ALDH1A3^low^, 159CON, 159ALDH1A1^low^, and 159ALDH1A3^low^. After 4 days, RNA, cell lysates, or cells were collected and (**A**,**B**) qRT-PCR, (**C**) immunoblotting, or (**D**) Aldefluor^®^ assays were performed to assess *ALDH1* gene expression, ALDH1 protein expression, and ALDH enzyme activity (respectively). Data represents the mean ± SEM. * = significantly different than respective siCON, 468CON, or 159CON scrambled control cells (*p* < 0.05).

siRNA was used to knockdown expression of two ALDH1 isozymes (ALDH1A1 and ALDH1A3) in MDA-MB-468 and SUM159 breast cancer cells and generate the following cell populations: 468CON, 468ALDH1A1^low^, 468ALDH1A3^low^, 159CON, 159ALDH1A1^low^, and 159ALDH1A3^low^. Knockdown of RNA and protein expression was confirmed by quantitative real-time polymerase chain reaction (RT-PCR) and immunoblotting respectively (Figure 2A–C).

There has been some debate over which ALDH1 isozyme is responsible for the ALDH enzymatic activity measured in the ALDEFLUOR^®^ assay. (StemCell Technologies, Vancouver, BC, Canada), with some groups suggesting that ALDH1A1 is responsible, while others believe that it is ALDH1A3 [27,29]. Compared to respective siRNA scrambled controls, we observed that 468ALDH1A3^low^ and 159ALDH1A3^low^ cell populations did demonstrate a significant decrease in ALDH activity (*p* < 0.001), while 468ALDH1A1^low^ and 159ALDH1A1^low^ cell populations did not exhibit a change in ALDH activity (*p* > 0.05) (Figure 2D). This data is further supported by the observation that *ALDH1A3* mRNA expression is higher than *ALDH1A1* mRNA expression in sorted ALDH^hi^ versus unsorted cell populations (Figure S2). Our data also supports previous observations by Marcato et al. (2011), and indicates that the ALDH1A3 isozyme is the major contributor to ALDH activity in breast cancer cells as measured by the ALDEFLUOR^®^ assay [27].

### 2.3. Decreased Expression of ALDH1A1 Reduces Breast Cancer Cell Proliferation, but Adhesion and Migration of Human Breast Cancer Cells Is Differentially Influenced by ALDH1A1 versus ALDH1A3 In Vitro

**Figure 3:**
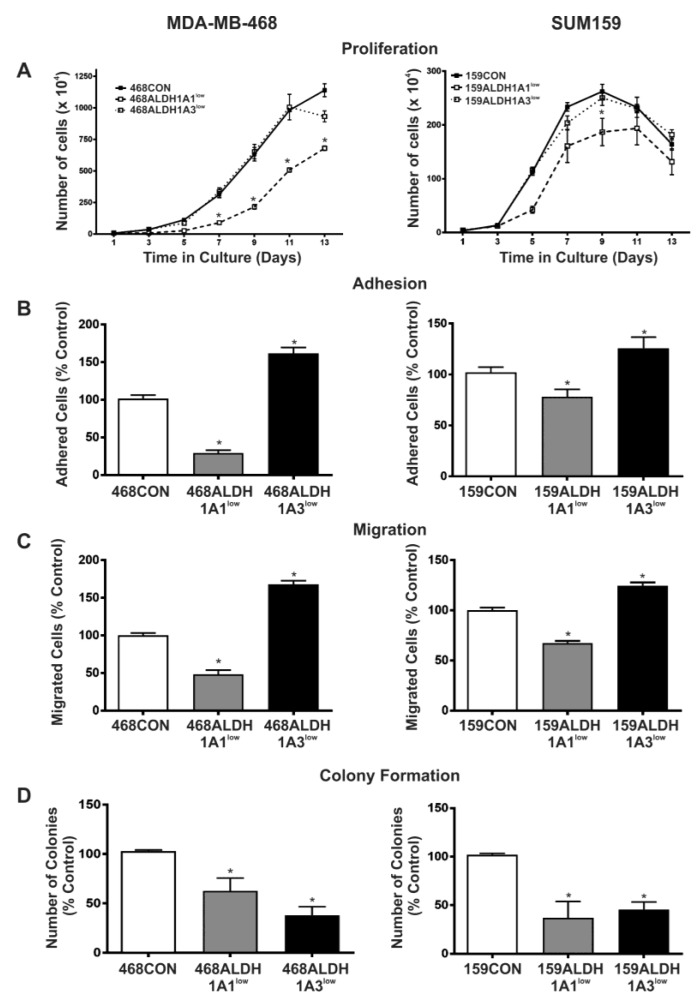
Decreased expression of ALDH1A1 reduces breast cancer cell proliferation, but adhesion and migration of human breast cancer cells is differentially influenced by ALDH1A1 versus ALDH1A3 in vitro. MDA-MB-468 (left panels) and SUM159 (right panels) human breast cancer cells were treated with control siRNA (siCON) or ALDH-specific siRNA (siALDH1A1 or siALDH1A3) for 96 h to generate the following cell lines: 468CON, 468ALDH1A1^low^, 468ALDH1A3^low^, 159CON, 159ALDH1A1^low^, 159ALDH1A3^low^. (**A**) Proliferation; (**B**) adhesion assays; (**C**) migration; and (**D**) colony formation. In all cases, data represents the mean ± SEM normalized to respective scrambled control. * = significantly different than respective scrambled control (*p* < 0.05).

Malignant breast cancer cell behavior in vitro was assessed in response to direct knockdown of ALDH1A1 or ALDH1A3 by siRNA (Figure 3). 468ALDH1A1^low^ and 159ALDH1A1^low^ cells demonstrated significantly decreased growth in normal culture relative to respective control cells (*p* < 0.05), whereas 468ALDH1A3^low^ and 159ALDH1A3^low^ cells showed no difference in proliferation compared to control cells. Lag times (time to reach exponential growth phase) were also observed to be longer for 468ALDH1A1^low^ and 159ALDH1A1^low^ cells versus respective control cells (9 days vs. 5 days for MDA-MB-468 cells; 5 days vs. 3 days for SUM159 cells) (Figure 3A). We next assessed the influence of ALDH1A1 and ALDH1A3 knockdown on breast cancer cell adhesion and migration in vitro (Figure 3B,C). 468ALDH1A1^low^ and 159ALDH1A1^low^ cells were observed to be significantly less adherent (Figure 3B), and less migratory (Figure 3C) than respective control cells (*p* < 0.05). In contrast, 468ALDH1A3^low^ and 159ALDH1A3^low^ cells were observed to be significantly more adherent and more migratory (Figure 3B,C) than respective control cells (*p* < 0.05), suggesting that adhesion and migration of human breast cancer cells is differentially influenced by ALDH1A1 versus ALDH1A3. Knockdown of either ALDH1A1 or ALDH1A3 resulted in reduced colony formation in soft agar relative to control cells (*p* < 0.05) (Figure 3D). It should be noted that the adhesion and migration assays (Figure 3B,C) are performed over time periods of 24 h or less when siRNA knockdown is strong. However, in the proliferation and colony-forming assays (Figure 3A,D), the studies extend well past when the knockdown would be expected to persist. This suggests that the influence of ALDH1 on proliferation and colony formation is an early but important effect that then has a “feed-forward” or downstream effect on the ability of breast cancer cells to proliferate or form established/persistent colonies.

### 2.4. Decreased Expression of ALDH1A1 and ALDH1A3 Reduces In Vivo Metastatic Ability of Breast Cancer Cells in the Chick Chorioallantoic Membrane (CAM) Assay

**Figure 4:**
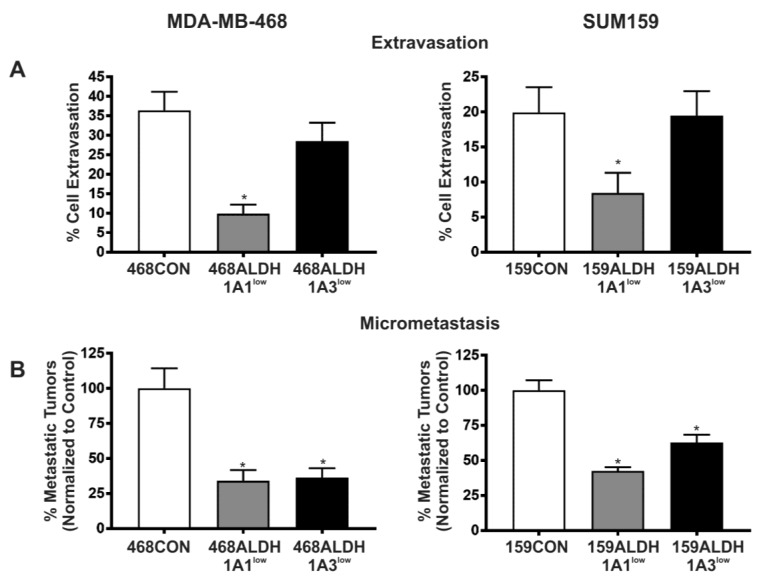
Decreased expression of ALDH1A1 and ALDH1A3 reduces in vivo metastatic ability of breast cancer cells in the chick CAM assay. GFP-labeled MDA-MB-468 or CMFDA-labeled SUM159 cell populations were transfected with 100 pmol (MDA-MB-468) or 400 pmol (SUM159) siRNA targeted towards ALDH1A1, ALDH1A3, or scrambled control using Lipofectamine to generate the following cell lines: 468CON, 468ALDH1A1^low^, 468ALDH1A3^low^, 159CON, 159ALDH1A1^low^, 159ALDH1A3^low^. After 4 days, 1 × 10^5^ (extravasation assay) or 2 × 10^5^ (micrometastasis assay) cells were injected into chicken embryos and (**A**) cell extravasation was observed after 24 h, or (**B**) micrometastatic formation was observed after 7days. Data represents the mean ± SEM normalized to control cells. * = significantly different than respective 468CON and 159CON cells (*p* < 0.05).

In order to assess the metastatic ability of ALDH-deficient cell populations in vivo, GFP-labeled MDA-MB-468 cell populations (468CON, 468ALDH1A1^low^, 468ALDH1A3^low^ cells) or CMFDA-labeled SUM159 cell populations (159CON, 159ALDH1A1^low^, 159ALDH1A3^low^ cells) were inoculated on the CAM of 9- or 12-day-old chicken embryos, and the percentage of breast cancer cell extravasation into the CAM and formation of micrometastases in the chicken embryo were analyzed (Figure 4). 468ALDH1A1^low^ and 159ALDH1A1^low^ cells demonstrated a significant decrease in extravasation compared to respective control cells (*p* < 0.05), whereas there was no significant difference observed in the extravasation of 468ALDH1A3^low^ or 159ALDH1A3^low^ cells compared to control (Figure 4A). In contrast, Both ALDH1A1^low^ and ALDH1A3^low^ cell populations from both MDA-MB-468 and SUM159 cell lines demonstrated a significant decrease in the number of micrometastatic tumors that were able to form compared to control (*p* < 0.05) (Figure 4B).

### 2.5. Decreased Expression of ALDH1A1 but Not ALDH1A3 Sensitizes Breast Cancer Cells to Chemotherapy and Radiation In Vitro

**Figure 5:**
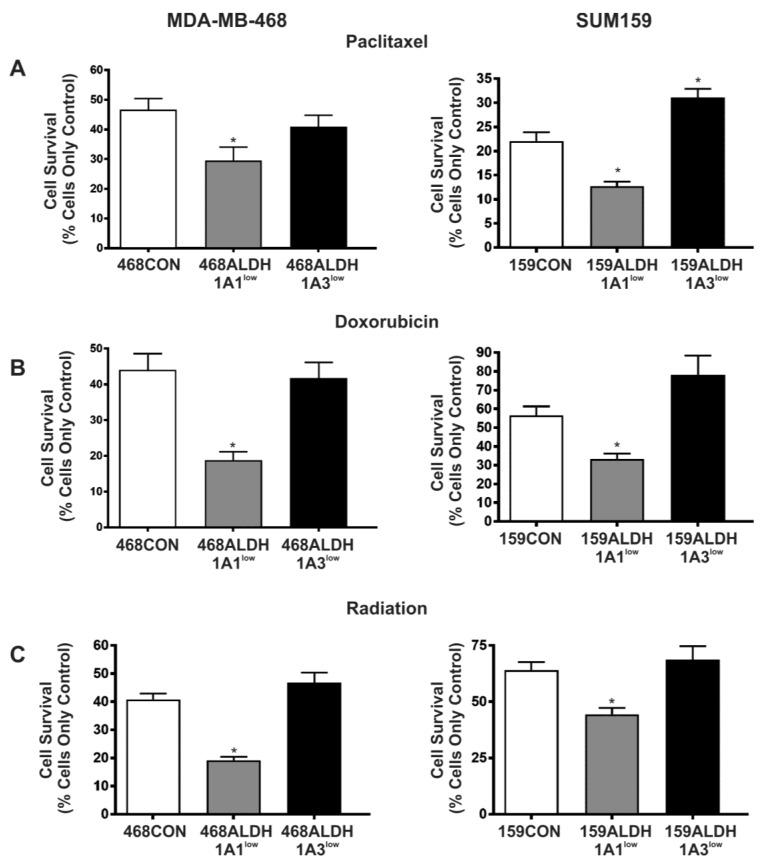
Decreased expression of ALDH1A1 but not ALDH1A3 sensitizes breast cancer cells to chemotherapy and radiation. MDA-MB-468 cells (left panels) and SUM159 cells (right panels) were treated with control siRNA (siCON) or ALDH-specific siRNA (ALDH1A1 or ALDH1A3) for 96 h to generate the following cell lines: 468CON, 468ALDH1A1^low^, 468ALDH1A3^low^, 159CON, 159ALDH1A1^low^, 159ALDH1A3^low^. Cell populations were treated with (**A**) paclitaxel (0.2 μg/mL), (**B**) doxorubicin (0.2 μg/mL), or (**C**) radiation (2 × 5Gy; MDA-MB-468 or 2 × 15Gy; SUM159). Data represents the mean ± SEM normalized to respective control cells. * = significantly different than respective 468CON or 159CON cells treated with paclitaxel, doxorubicin, or radiation (*p* < 0.01).

Finally, we have previously observed that breast cancer cells with high ALDH activity and CD44 expression (ALDH^hi^CD44^+^ phenotype) are significantly more resistant to chemotherapy and radiation therapy, and that this therapy resistance may occur, at least in part, via ALDH1-dependent mechanisms [9]. Taken together with the known role of ALDH activity in cellular self-protection and detoxification [30], we hypothesized that a siRNA-mediated reduction in ALDH1 expression would sensitize MDA-MB-468 and SUM159 cells to chemotherapy and radiation. We observed that knockdown of ALDH1A1 caused a significant sensitization of both MDA-MB-468 and SUM159 cells to paclitaxel (Figure 5A), doxorubicin (Figure 5B), and radiation therapy (Figure 5C) (*p* < 0.05). In contrast, ALDH1A3 knockdown did not reduce therapy resistance compared to control cells (Figure 5A–C).

## 3. Discussion

Breast cancer is a leading cause of death in women, primarily due to ineffective treatment of metastatic disease [1,2]. Our group has previously demonstrated that stem-like ALDH^hi^CD44^+^ cells play a key role in breast cancer metastasis [6] and are highly resistant to chemotherapy and radiation compared to their ALDH^low^CD44^−^ counterparts, potentially as a result of ALDH-dependent mechanisms [9]. Additionally, it has been shown that ALDH1 expression is correlated with early recurrence, worse prognosis, and a higher incidence of metastasis in breast cancer patients [7,20,21,27]. While this suggests that ALDH is an important player in breast cancer metastasis; the actual functional contribution of ALDH1 (in particular its isozymes ALDH1A1 and ALDH1A3) in breast cancer metastasis requires further elucidation, and this was the goal of the current study.

Although the Aldefluor^®^ assay is often used to isolate ALDH^hi^ cancer cells [6,7,8,9,20,31,32], the specific ALDH isozymes that contribute to this activity remain a subject of debate. In this assay, cells are incubated in a buffer containing a fluorescent aldehyde substrate (bodipy-aminoacetylaldehyde). The aminoacetylaldehyde is taken up into the cells via passive diffusion. Once inside the cell, intracellular ALDH oxidizes the aminoacetylaldehyde into aminoacetate, which is negatively charged, and therefore retained inside the cell, causing the cells to fluoresce [32]. When ALDH1A1 was knocked down in both MDA-MB-468 and SUM159 cell lines, there was no observable change in ALDH activity as measured by the Aldefluor^®^ assay; however, when ALDH1A3 was knocked down, there was an approximate 50% reduction in ALDH activity measured by the Aldefluor^®^ assay. This is consistent with breast cancer studies done by Marcato et al. (2011), who observed that ALDH1A3 knockdown was better correlated with a decrease in Aldefluor^®^ activity compared to ALDH1A1 and ALDH2 [27]. Additional studies have reported that ALDH1A1, ALDH7A1, ALDH2 and/or ALDH1A2 are responsible for driving Aldefluor^®^ activity in other tumor types [32,33,34], indicating that the ALDH isoform(s) responsible for Aldefluor^®^ activity may be tumor-specific. Furthermore, in the present study, even after ALDH1A3 knockdown, there was still approximately 50% normal ALDH activity, indicating that other ALDH isozymes might be involved in the context of breast cancer. Taken together, these results suggest that many ALDH isozymes may contribute to the ALDH activity measured by the Aldefluor^®^ assay, and potentially that different isozymes may contribute to ALDH activity in different tumor types.

We previously reported that ALDH^hi^CD44^+^ cells demonstrated enhanced proliferation, adhesion, and migration [6]. Additional work in lung and liver cancer cells has suggested that a decrease in ALDH expression can result in a decrease in proliferation [35,36,37]. In the current study, we treated breast cancer cells with DEAB (a direct competitive substrate of ALDH [28]) and observed a decrease in cell proliferation, as well as in adhesion and migration in vitro compared to control cells, suggesting that ALDH may potentially contribute to these processes. In order to determine whether ALDH1 isozymes were also involved in these processes, we used siRNA to specifically knockdown ALDH1A1 or ALDH1A3 and observed that ALDH1A1^low^ cells demonstrated decreased proliferation, adhesion, and migration in vitro. In contrast, cells in which ALDH1A3 had been knocked down showed no change in proliferation and in fact demonstrated increased levels of adhesion and migration in vitro.

ALDH1 expression has been clinically correlated with an increased incidence of metastasis [7,20,27]. We used the chick CAM assay to elucidate whether ALDH1A1 and/or ALDH1A3 functionally contributed to metastasis. Cells with decreased ALDH1A1 expression demonstrated decreased abilities to invade/extravasate; whereas cells with decreased ALDH1A3 expression demonstrated no change in invasive capabilities compared to control cells in vivo. However, in terms of the actual formation of metastases in vivo; both ALDH1A1^low^ and ALDH1A3^low^ cells demonstrated a decrease in metastatic potential, with an approximate 50% reduction in the number of micrometastases that were able to form in the chick CAM compared to control cells.

Finally, we have previously observed that that ALDH^hi^CD44^+^ cells demonstrate high levels of therapy resistance, and that pre-treatment targeting of ALDH activity using DEAB or ATRA can sensitize these resistant cells to both anthracycline and taxane chemotherapy, as well as radiation [9]. In the current study, we directly targeted specific ALDH1 isozymes using siRNA and tested the effect on therapy response. Notably, when ALDH1A1 expression was decreased, there was a significant sensitization of the cancer cells to both chemotherapy and radiation. Cells with decreased ALDH1A3 expression, however, showed no change in therapy resistance to either chemotherapy or radiation. These results suggest that the ALDH1A1 isozyme is an important contributor to therapy resistance in breast cancer cells, not only to cyclophosphamide chemotherapy (as previously reported [13,38]), but also to other classes of chemotherapy and radiotherapy.

Our study is the first in the literature to systematically examine the functional roles of ALDH1 isozymes on individual breast cancer cell behaviors that collectively contribute to the metastatic process. The combined in vitro and in vivo data presented in this study suggests that ALDH1A1 and ALDH1A3 both contribute functionally to various steps in the breast cancer metastatic cascade; however, they may do so in different ways (summarized in Table 1). For example, it appears that ALDH1A1 may mediate the adhesion, migration, extravasation, and initial colonization steps; whereas ALDH1A3 may only participate in colonization and sustainment of metastatic growth. This data both supports and contradicts previous work by Marcato et al. (2011), who reported that ALDH1A3 and not ALDH1A1 correlated with metastatic disease in breast cancer patients [27]. More recent work by this group led to the observation that overexpression of ALDH1A3 in MDA-MB-231 human breast cancer cells increases in vitro invasion and in vivo primary tumor growth and lung metastasis in mice, likely due to changes in RA signaling [39]. Although they observed that knockdown of either ALDH1A1 or ALDH1A3 in MDA-MB-231 cells did not have an effect on malignant behavior, this was not surprising given that this cell line has very low levels of these isozymes to begin with [27]. In contrast, it was somewhat surprising that their knockdown of ALDH1A1 in MDA-MB-468 cells (one of the cell lines used in the present study) actually increased primary tumor growth in mice, which is somewhat in contrast with our observed reduction in proliferation, colony-formation, and in vivo metastasis data presented in the current study. Overall, Marcato et al. [39] observed cell line-specific differences with regards to ALDH1A3 function in malignancy and metastasis. In contrast, our data shows that knockdown of ALDH1A1 consistently reduces most steps in the metastatic cascade except for basic proliferation in two different human breast cancer cell lines with different genetic backgrounds and differing metastatic ability. These experimental findings are supported by clinical data, which demonstrates that ALDH1A1 expression is often associated with worse prognosis in breast and other cancers [7,20,24,40,41,42,43].

Overall, the results of this study support the concept that ALDH1 plays a functional role in both breast cancer metastasis and therapy resistance; although the ALDH1A1 and ALDH1A3 isozymes seemed to contribute to these behaviors in different ways. In order to determine the underlying reasons for the differential influence of ALDH1 on different malignant behaviors, in-depth mechanistic studies will need to be carried out in the future. In addition, the observation that ALDH1A3 knockdown only caused a 50% reduction in ALDH activity suggests that other ALDH isozymes must be involved in Aldefluor^®^ activity in breast cancer cells. It would therefore be interesting in the future to determine the functional role of other ALDH isozymes in breast cancer metastasis (i.e., ALDH7A1, ALDH1A2, and/or ALDH2) [33], as well as to assess corresponding changes in genes, transcription factors, and epigenetic modifiers that may ultimately be driving the process of metastasis. Elucidation of the mechanisms by which ALDH1A1, ALDH1A3 and other ALDH isozymes contribute to disease progression could have potentially important implications for the management and treatment of breast cancer in the future. Furthermore, additional investigation of ALDH1A1-specific therapy resistance mechanisms is required, and translating this knowledge into the clinic through development of either a direct, specific ALDH1A1 inhibitor or an ALDH1A1-related inhibitor that is safe for human use could have important implications for the management of both primary and metastatic breast cancer. Finally, it is well known that treating breast cancer before metastasis is observed (i.e., in the adjuvant setting) is significantly correlated with better patient survival [6,9,44]. Given that ALDH1 has been both correlated with metastatic disease and shown to functionally contribute to metastasis, it may be beneficial to use assessment of ALDH1 expression in the primary tumor as a clinical tool for identifying breast cancer patients with a high risk of metastasis and stratifying them for aggressive therapy to prevent disease recurrence or progression.

## 4. Materials and Methods

### 4.1. Cell Culture, Reagents, and Therapy Conditions

MDA-MB-468 cells were a kind gift from Dr. Janet Price, M.D. Anderson Cancer Center, (Houston, TX, USA) [45], and were maintained in αMEM +10% fetal bovine serum (FBS). The 468 subline expressing green fluorescent protein (GFP) was generated previously as described [46]. SUM159 cells [47] were obtained from Asterand (Detriot, MI, USA) and maintained in Hams: F12 + 5% FBS. CellTracker™ 5-chloromethylfluorescein diacetate (CMFDA; Invitrogen, Carlsbad, CA, USA) was used to label SUM159 cells for the CAM assay. All cell lines were authenticated via third party testing of 9 short tandem repeat (STR) loci on 11 April 2103. (CellCheck, RADIL, Columbia, MO, USA). All media was obtained from Invitrogen. FBS was obtained from Sigma (St. Louis, MO, USA). Tissue culture plastic was obtained from NUNC (Roskilde, Denmark).

All-*trans* retinoic acid (ATRA) and diethylamino-benzaldehyde DEAB (Sigma) were constituted in 100% ethanol and diluted in either Hams:F12 (SUM159 cells) or α-MEM (MDA-MB-468 cells) at 5 μM (ATRA) or 100 μM (DEAB). Doxorubicin (Novopharm Limited, Toronto, ON, Canada) and paclitaxel (Biolyse Pharma Corporation, St. Catherines, ON, Canada) were diluted in either Hams: F12 or α-MEM to the concentrations noted below. Radiation was administered at the doses noted below using a Cobalt-60 irradiator (Theratron 60, Atomic Energy of Canada Limited, Chalk River, ON, Canada). All treatment doses were selected based on LC_50_ values determined in previous experiments [9].

### 4.2. Cell Proliferation Assays

Breast cancer cells were counted and plated at a density of 5.0 × 10^4^ cells/60 mm plate (*n* = 3 per time point) and maintained in regular growth media. Every 48 h for 14 days, cultures (*n* = 3) were trypsinized and counted using a hemocytometer. Doubling time of each cell population was estimated during the exponential growth phase according to *T*_d_ = 0.693*t*/ln (*N*_t_/*N*_0_), where *t* is time (in hours), *N*_t_ is the cell number at time *t*, and *N*_0_ is the cell number at initial time.

### 4.3. Cell Adhesion Assays

Breast cancer cells were plated onto sterile 96-well non-tissue culture plates (Titertek, Flow Laboratories Inc.; McLean, VA, USA) that had been treated with one of: 20 μg/mL of human laminin (Sigma; SUM159 cells), 5 μg/mL of human vitronectin (Sigma; MDA-MB-468 cells), or PBS (negative control), using 1 × 10^4^ cells/well (*n* = 3) for each cell population. Laminin and vitronectin were chosen based on previous experiments in our laboratory that have demonstrated that SUM159 and MDA-MB-468 cells differentially express integrin receptors for vitronectin and laminin respectively [48,49]. Cells were allowed to adhere for 5 h, after which non-adhered cells were rinsed away. Adhered cells were fixed with 2% gluteraldehyde and stained using Harris’ hematoxylin. Five high powered fields (HPF) (200×) were counted for each well, and mean numbers of adhered cells/field were calculated and normalized to control cell populations.

### 4.4. Cell Migration Assays

Transwell plates (8 μm pore size, 6.5 mm; Becton Dickinson; Franklin Lakes, NJ, USA) were coated with 6 μg/well of gelatin (Sigma) [50,51]. Chemoattractant (5% FBS) or control (0.01% BSA) media was placed in the bottom portion of each well. For each cell population, 5 × 10^4^ cells were plated on top of the transwells. After 24 h, the upper transwell was removed, inverted, fixed with 1% gluteraldehyde, and stained with Harris’ hematoxylin. A cotton swab was used to carefully remove non-migrated cells on the inner surface of the transwell. For each well, five HPF were counted and mean numbers of migrated cells/field were calculated and normalized to control cell populations.

### 4.5. Colony Forming Assays

Dishes (60 mm) were coated with 1% agarose (Bioshop; Burlington, ON, Canada) in normal growth media and allowed to solidify for 1 hr. Breast cancer cell suspensions (1.0 × 10^4^ cells/60 mm plate) were prepared using 0.6% agarose in normal growth media and plated on top of the base agarose base layer (*n* = 4 for each time point). Normal growth media was added on top of the cell layer and changed every 3–4 days for 4 weeks, after which the media was removed and plates were fixed in 10% neutral-buffered formalin (EM Sciences, Gladstone, NJ, USA). For each dish, 5 HPF were counted and mean number of colonies per field were calculated and normalized to control cell populations.

### 4.6. siRNA Knockdown of ALDH1A1 and ALDH1A3

ON-TARGET plus SMART pool small interfering RNAs (siRNA) (Dharmacon Thermo Scientific, Lafayette, CO, USA) were used to transiently transfect human ALDH1A1 and ALDH1A3 into MDA-MB-468 and SUM149 cells. All siRNAs were suspended in sterile RNAse-free water at a concentration of 25 μM. Scrambled control (20–50 μL/mL), ALDH1A1 (20 μL/mL), ALDH1A3 (50 μL/mL) siRNAs and Lipofectamine RNAiMax reagent (20 μL/mL; Invitrogen) were diluted into serum-free Opti-MEM (Invitrogen). Lipofectamine and siRNA concentrations were determined based on preliminary experiments which indicated the greatest knockdown of the proteins of interest [49]. The transfections yielded the following cell populations used in further experiments: 468CON, 468ALDH1A1^low^, 468ALDH1A3^low^, 159CON, 159ALDH1A1^low^, and 159ALDH1A3^low^.

### 4.7. RNA Isolation and Quantitative RT-PCR

Total RNA was extracted using TRIzol (Invitrogen) according to the manufacturer’s protocol. Total RNA was reverse transcribed using Superscript III (Invitrogen) and the Eppendorf Mastercycler Gradient (Eppendorf, Hamburg, Germany). Primers and cycling conditions used for *ALDH1A1*, *ALDH1A3*, and *GAPDH* are provided in Table 2. Relative quantification of *ALDH1A1* and *ALDH1A3* gene expression in MDA-MB-468 and SUM159 breast cancer cells was determined by quantitative PCR using Brilliant^®^ II SYBR^®^ Green qPCR Low ROX Master Mix (Agilent Technologies, Eugene, OR, USA) and the delta Ct method. *GAPDH* was used for normalization.

### 4.8. Immunoblotting

Cell lysates were extracted and protein (10 μg) was subjected to sodium dodecyl sulfate polyacrylamide gel electrophoresis (SDS-PAGE, 12%) and transferred onto polyvinylidene difluoride membranes (PVDF; Immobilon™, Millipore; Bedford, MA, USA). Blocking and antibody dilution was done using 5% skim milk in Tris-buffered saline with 0.1% Tween-20 (TBST). Anti-human primary antibodies included mouse monoclonal ALDH1A1 (clone IG6; 1:1000) and rabbit polyclonal ALDH1A3 (1:500) (Abcam, Cambridge, MA, USA). Secondary antibodies included goat anti-mouse and mouse anti-rabbit antibodies conjugated to horseradish peroxidase (Calbiochem, Gibbstown, NJ, USA) (1:2000). Protein expression was visualized using Amersham ECL Plus (GE Healthcare, Baie d’Urfe, QC, Canada) using β-actin (Sigma, 1:5000) as a loading control.

### 4.9. ALDEFLUOR^®^ Assay

The ALDEFLUOR^®^ assay (StemCell Technologies, Vancouver, BC, Canada) was used to assess ALDH activity as described previously [52,53,54]. Briefly, cells were harvested, placed in ALDEFLUOR^®^ assay buffer (2 × 10^6^/mL), and incubated with ALDEFLUOR^®^ substrate for 45 min at 37 °C to allow substrate conversion. As a negative control for all experiments, an aliquot of ALDEFLUOR^®^-stained cells was immediately quenched with 1.5-mM diethylaminobenzaldehyde (DEAB), a specific ALDH inhibitor. Cells were analyzed using the green fluorescence channel (FL1) on a Beckman Coulter EPICS XL-MCL flow cytometer.

### 4.10. Chick Embryo Chorioallantoic Membrane (CAM) Assay

For assessment of in vivo extravasation and metastasis, chick embryo chorioallantoic membrane (CAM) assays were used as described previously [55,56]. Briefly, fertilized chicken eggs (McKinley Hatchery, St. Mary’s, ON, Canada) were removed from their shell, placed in covered dishes, and maintained *ex ovo* at 37 °C with 90% humidity. Embryos were used at day 9 (micrometastasis assay) and day 12 (extravasation assay). Green-fluorescent protein (GFP) labeled MDA-MB-468 or CellTracker™ CMFDA-labeled SUM159 cell populations were injected intravenously (i.v.) into the CAM as described previously [55,56] using 1 × 10^5^ (extravasation assay) or 2 × 10^5^ (micrometastasis assay) cells/egg (*n* = 8–17 eggs per treatment group). For the extravasation assay, a portion of the CAM was sectioned off using aluminum foil and the number of cells arrested in the sectioned-off area was manually counted using a fluorescence microscope at 20× magnification. Embryos were then returned to the incubator for 24 h, after which time the number of extravasated cells in the sectioned off area were manually counted using a fluorescence microscope. Percent extravasation was calculated by dividing the number of initial cells by the number of successfully extravasated cells in the CAM. For the micrometastasis assay, embryos were returned to the incubator for 7 days after cell injection to allow the formation of metastases. After 7 days, the number of micrometastatic tumors that developed following the i.v. injection were manually counted using a fluorescence microscope at 4× magnification.

### 4.11. Chemotherapy and Radiation Treatment

Cell populations were plated at a density of 5 × 10^5^ cells in 6-well plates (*n* = 3/treatment group) and maintained in normal growth medium for 24 h. Cells were then treated with either normal media alone (control), chemotherapy (paclitaxel (0.2 μM); doxorubicin (0.4 μM)), or radiation (2 × 5Gy, MDA-MB-468; or 2 × 15Gy, SUM159) and cultured for a further 72 h. Cells were then harvested and viable cells were quantified using trypan blue exclusion and manual counting on a hemocytometer using light microscopy.

### 4.12. Statistical Analysis

All experiments were performed following at least three separate siRNA transfections with at least 3 biological replicates included within each experiment. In all cases, quantitative data was compiled from all experiments. Statistical analysis was performed using GraphPad Prism 4.0 software© (San Diego, CA, USA) using either *t*-test (for comparison between 2 groups) or analysis of variance (ANOVA) with Tukey post-test (for comparison between more than 2 groups) when groups passed both a normality test and an equal variance test. When this was not the case, the Mann-Whitney Rank-Sum test was used. Unless otherwise noted, data is presented as the mean ± SEM. In all cases, *p* values of ≤0.05 were regarded as being statistically significant.

## Figures and Tables

**Table 1:** Summary of functional consequences of ALDH1A1 and ALDH1A3 knockdown in MDA-MB-468 and SUM159 human breast cancer cells.

Functional Behavior/Activity	ALDH1A1 Knockdown	ALDH1A3 Knockdown
ALDH Activity (Aldeflour)	No effect	↓
Proliferation	↓	No effect
Adhesion	↓	↑
Migration	↓	↑
Colony Formation	↓	↓
Extravasation	↓	No effect
Metastasis	↓	↓
Therapy Resistance	↓	No effect

↑ = increase in respective functional behavior/activity; ↓ = decrease in respective functional behavior/activity.

**Table 2:** Primers and qPCR conditions.

Gene	Primer Sequence	qPCR Cycling Conditions	Number of Cycles	Product Size (bp)
*ALDH1A1*	Fwd: 5′-CGT TGG TTA TGC TCA TTT GGA A-3′ Rev: 5′-TGA TCA ACT TGC CAA CCT CTG T-3′	60 s 55 °C 60 s 72 °C 60 s 95 °C	45	22 bp
*ALDH1A3*	Fwd: 5′-ATG TGG GAA AAC CCC CTG TG-3′ Rev: 5′-GAA TGG TCC CAC CTT CAC CT-3′	60 s 57 °C 60 s 72 °C 60 s 95 °C	45	20 bp
*GAPDH*	Fwd: 5′-CAT GTT CGT CAT GGG TGT GAA CCA-3′ Rev: 5′-ATG GCA TGG ACT GTG GTC ATG AGT -3′	45 s 60 °C 45 s 72 °C 60 s 95 °C	40	24 bp

## Supplementary Materials

Supplementary materials can be found at www.mdpi.com/1422-0067/18/10/2039/s1.

## Abbreviations

ALDH	Aldehyde dehydrogenase
ANOVA	Analysis of variance
APL	Acute promyelocytic leukemia
ATRA	All-*trans* retinoic acid
BSA	Bovine serum albumin
CAM	Choroiallantoic membrane assay
CD	Cluster of differentiation
CMFDA	5-chloromethylfluorescein diacetate
CYP	Cytochrome P450
DEAB	Diethylaminobenzaldehyde
ECL	Enhanced chemiluminescence
ER	Estrogen receptor
EtOH	Ethanol
FBS	Fetal bovine serum
GAPDH	Glyceraldehyde 3-phosphate dehydrogenase
GFP	Green fluorescent protein
Gy	Gray
HPF	High-powered field
LC_50_	Lethal concentration (50%)
NAD(P)	Nicotinamide adenine dinucleotide phosphate
PBS	Phosphate buffered saline
PCR	Polymerase chain reaction
PR	Progesterone receptor
PVDF	Polyvinylidene fluoride
RA	Retinoic acid
RT	Reverse transcriptase
SDS-PAGE	Sodium dodecyl sulfate polyacrylamide gel electrophoresis
SEM	Standard error of the mean
siRNA	Small interfering RNA
STR	Short tandem repeat
TBST	Tris-buffered saline + Tween-20

## References

[1] Chambers A.F., Groom A.C., MacDonald I.C. (2002). Dissemination and growth of cancer cells in metastatic sites. Nat. Rev. Cancer.

[2] Siegel R.L., Miller K.D., Jemal A. (2017). Cancer statistics, 2017. CA Cancer J. Clin..

[3] Luzzi K.J., MacDonald I.C., Schmidt E.E., Kerkvliet N., Morris V.L., Chambers A.F., Groom A.C. (1998). Multistep nature of metastatic inefficiency: Dormancy of solitary cells after successful extravasation and limited survival of early micrometastases. Am. J. Pathol..

[4] Weiss L. (1990). Metastatic inefficiency. Adv. Cancer Res..

[5] Goss P., Allan A.L., Rodenhiser D.I., Foster P.J., Chambers A.F. (2008). New clinical and experimental approaches for studying tumor dormancy: Does tumor dormancy offer a therapeutic target?. APMIS.

[6] Croker A.K., Goodale D., Chu J., Postenka C., Hedley B.D., Hess D.A., Allan A.L. (2009). High aldehyde dehydrogenase and expression of cancer stem cell markers selects for breast cancer cells with enhanced malignant and metastatic ability. J. Cell. Mol. Med..

[7] Charafe-Jauffret E., Ginestier C., Iovino F., Tarpin C., Diebel M., Esterni B., Houvenaeghel G., Extra J.M., Bertucci F., Jacquemier J. (2010). Aldehyde dehydrogenase 1-positive cancer stem cells mediate metastasis and poor clinical outcome in inflammatory breast cancer. Clin. Cancer Res..

[8] Charafe-Jauffret E., Ginestier C., Iovino F., Wicinski J., Cervera N., Finetti P., Hur M.H., Diebel M.E., Monville F., Dutcher J. (2009). Breast cancer cell lines contain functional cancer stem cells with metastatic capacity and a distinct molecular signature. Cancer Res..

[9] Croker A.K., Allan A.L. (2012). Inhibition of aldehyde dehydrogenase (ALDH) activity reduces chemotherapy and radiation resistance of stem-like ALDHhiCD44(+) human breast cancer cells. Breast Cancer Res. Treat..

[10] Rodriguez-Torres M., Allan A.L. (2016). Aldehyde dehydrogenase as a marker and functional mediator of metastasis in solid tumors. Clin. Exp. Metastasis.

[11] Pors K., Moreb J.S. (2014). Aldehyde dehydrogenases in cancer: An opportunity for biomarker and drug development?. Drug Discov. Today.

[12] Collins C.A., Watt F.M. (2008). Dynamic regulation of retinoic acid-binding proteins in developing, adult and neoplastic skin reveals roles for β-catenin and notch signalling. Dev. Biol..

[13] Sladek N.E. (2003). Human aldehyde dehydrogenases: Potential pathological, pharmacological, and toxicological impact. J. Biochem. Mol. Toxicol..

[14] Fenaux P., Castaigne S., Dombret H., Archimbaud E., Duarte M., Morel P., Lamy T., Tilly H., Guerci A., Maloisel F. (1992). All-transretinoic acid followed by intensive chemotherapy gives a high complete remission rate and may prolong remissions in newly diagnosed acute promyelocytic leukemia: A pilot study on 26 cases. Blood.

[15] Sanz M.A., Lo-Coco F. (2011). Modern approaches to treating acute promyelocytic leukemia. J. Clin. Oncol..

[16] Elizondo G., Corchero J., Sterneck E., Gonzalez F.J. (2000). Feedback inhibition of the retinaldehyde dehydrogenase gene *ALDH1* by retinoic acid through retinoic acid receptor alpha and ccaat/enhancer-binding protein β. J. Biol. Chem..

[17] Ozeki M., Shively J.E. (2008). Differential cell fates induced by all-*trans* retinoic acid-treated HL-60 human leukemia cells. J. Leukoc. Biol..

[18] Ginestier C., Wicinski J., Cervera N., Monville F., Finetti P., Bertucci F., Wicha M.S., Birnbaum D., Charafe-Jauffret E. (2009). Retinoid signaling regulates breast cancer stem cell differentiation. Cell Cycle.

[19] Tanei T., Morimoto K., Shimazu K., Kim S.J., Tanji Y., Taguchi T., Tamaki Y., Noguchi S. (2009). Association of breast cancer stem cells identified by aldehyde dehydrogenase 1 expression with resistance to sequential paclitaxel and epirubicin-based chemotherapy for breast cancers. Clin. Cancer Res..

[20] Ginestier C., Hur M.H., Charafe-Jauffret E., Monville F., Dutcher J., Brown M., Jacquemier J., Viens P., Kleer C.G., Liu S. (2007). ALDH1 is a marker of normal and malignant human mammary stem cells and a predictor of poor clinical outcome. Cell Stem Cell.

[21] Zhong Y., Lin Y., Shen S., Zhou Y., Mao F., Guan J., Sun Q. (2013). Expression of ALDH1 in breast invasive ductal carcinoma: An independent predictor of early tumor relapse. Cancer Cell Int..

[22] Kida K., Ishikawa T., Yamada A., Shimada K., Narui K., Sugae S., Shimizu D., Tanabe M., Sasaki T., Ichikawa Y. (2016). Effect of aldh1 on prognosis and chemoresistance by breast cancer subtype. Breast Cancer Res. Treat..

[23] Miyoshi Y., Shien T., Ogiya A., Ishida N., Yamazaki K., Horii R., Horimoto Y., Masuda N., Yasojima H., Inao T. (2016). Differences in expression of the cancer stem cell marker aldehyde dehydrogenase 1 among estrogen receptor-positive/human epidermal growth factor receptor type 2-negative breast cancer cases with early, late, and no recurrence. Breast Cancer Res. BCR.

[24] Khoury T., Ademuyiwa F.O., Chandrasekhar R., Jabbour M., Deleo A., Ferrone S., Wang Y., Wang X. (2012). Aldehyde dehydrogenase 1A1 expression in breast cancer is associated with stage, triple negativity, and outcome to neoadjuvant chemotherapy. Mod. Pathol..

[25] Zhou L., Jiang Y., Yan T., Di G., Shen Z., Shao Z., Lu J. (2010). The prognostic role of cancer stem cells in breast cancer: A meta-analysis of published literatures. Breast Cancer Res. Treat..

[26] Visus C., Wang Y., Lozano-Leon A., Ferris R.L., Silver S., Szczepanski M.J., Brand R.E., Ferrone C.R., Whiteside T.L., Ferrone S. (2011). Targeting ALDH(bright) human carcinoma-initiating cells with ALDH1A1-specific CD8(+) T cells. Clin. Cancer Res..

[27] Marcato P., Dean C.A., Pan D., Araslanova R., Gillis M., Joshi M., Helyer L., Pan L., Leidal A., Gujar S. (2011). Aldehyde dehydrogenase activity of breast cancer stem cells is primarily due to isoform ALDH1A3 and its expression is predictive of metastasis. Stem Cells.

[28] Koppaka V., Thompson D.C., Chen Y., Ellermann M., Nicolaou K.C., Juvonen R.O., Petersen D., Deitrich R.A., Hurley T.D., Vasiliou V. (2012). Aldehyde dehydrogenase inhibitors: A comprehensive review of the pharmacology, mechanism of action, substrate specificity, and clinical application. Pharmacol. Rev..

[29] Chute J.P., Muramoto G.G., Whitesides J., Colvin M., Safi R., Chao N.J., McDonnell D.P. (2006). Inhibition of aldehyde dehydrogenase and retinoid signaling induces the expansion of human hematopoietic stem cells. Proc. Natl. Acad. Sci. USA.

[30] Vasiliou V., Nebert D.W. (2005). Analysis and update of the human aldehyde dehydrogenase (ALDH) gene family. Hum. Genom..

[31] Moreb J.S., Baker H.V., Chang L.J., Amaya M., Lopez M.C., Ostmark B., Chou W. (2008). Aldh isozymes downregulation affects cell growth, cell motility and gene expression in lung cancer cells. Mol. Cancer.

[32] Moreb J.S., Zucali J.R., Ostmark B., Benson N.A. (2007). Heterogeneity of aldehyde dehydrogenase expression in lung cancer cell lines is revealed by aldefluor flow cytometry-based assay. Cytom. Part B Clin. Cytom..

[33] Hoogen C., Horst G., Cheung H., Buijs J.T., Pelger R.C.M., Pluijm G. (2011). The aldehyde dehydrogenase enzyme 7A1 is functionally involved in prostate cancer bone metastasis. Clin. Exp. Metastasis.

[34] Moreb J.S., Ucar D., Han S., Amory J.K., Goldstein A.S., Ostmark B., Chang L.J. (2012). The enzymatic activity of human aldehyde dehydrogenases 1A2 and 2 (ALDH1A2 and ALDH2) is detected by aldefluor, inhibited by diethylaminobenzaldehyde and has significant effects on cell proliferation and drug resistance. Chem. Biol. Interact..

[35] Canuto R.A., Muzio G., Salvo R.A., Maggiora M., Trombetta A., Chantepie J., Fournet G., Reichert U., Quash G. (2001). The effect of a novel irreversible inhibitor of aldehyde dehydrogenases 1 and 3 on tumour cell growth and death. Chem. Biol. Interact..

[36] Muzio G., Maggiora M., Paiuzzi E., Oraldi M., Canuto R.A. (2012). Aldehyde dehydrogenases and cell proliferation. Free Radic. Biol. Med..

[37] Muzio G., Trombetta A., Martinasso G., Canuto R.A., Maggiora M. (2003). Antisense oligonucleotides against aldehyde dehydrogenase 3 inhibit hepatoma cell proliferation by affecting map kinases. Chem. Biol. Interact..

[38] Moreb J.S., Mohuczy D., Ostmark B., Zucali J.R. (2007). Rnai-mediated knockdown of aldehyde dehydrogenase class-1A1 and class-3A1 is specific and reveals that each contributes equally to the resistance against 4-hydroperoxycyclophosphamide. Cancer Chemother. Pharmacol..

[39] Marcato P., Dean C.A., Liu R.Z., Coyle K.M., Bydoun M., Wallace M., Clements D., Turner C., Mathenge E.G., Gujar S.A. (2015). Aldehyde dehydrogenase 1A3 influences breast cancer progression via differential retinoic acid signaling. Mol. Oncol..

[40] Li T., Su Y., Mei Y., Leng Q., Leng B., Liu Z., Stass S.A., Jiang F. (2010). ALDH1A1 is a marker for malignant prostate stem cells and predictor of prostate cancer patients’ outcome. Lab. Investig..

[41] Li X., Wan L., Geng J., Wu C.L., Bai X. (2012). Aldehyde dehydrogenase 1A1 possesses stem-like properties and predicts lung cancer patient outcome. J. Thorac. Oncol..

[42] Morimoto K., Kim S.J., Tanei T., Shimazu K., Tanji Y., Taguchi T., Tamaki Y., Terada N., Noguchi S. (2009). Stem cell marker aldehyde dehydrogenase 1-positive breast cancers are characterized by negative estrogen receptor, positive human epidermal growth factor receptor type 2, and high ki67 expression. Cancer Sci..

[43] Neumeister V., Agarwal S., Bordeaux J., Camp R.L., Rimm D.L. (2010). In situ identification of putative cancer stem cells by multiplexing ALDH1, CD44, and cytokeratin identifies breast cancer patients with poor prognosis. Am. J. Pathol..

[44] Cristofanilli M. (2012). Advancements in the treatment of metastatic breast cancer (MBC): The role of ixabepilone. J. Oncol..

[45] Price J.E., Polyzos A., Zhang R.D., Daniels L.M. (1990). Tumorigenicity and metastasis of human breast carcinoma cell lines in nude mice. Cancer Res..

[46] Vantyghem S.A., Allan A.L., Postenka C.O., Al-Katib W., Keeney M., Tuck A.B., Chambers A.F. (2005). A new model for lymphatic metastasis: Development of a variant of the MDA-MB-468 human breast cancer cell line that aggressively metastasizes to lymph nodes. Clin. Exp. Metastasis.

[47] Flanagan L., Van Weelden K., Ammerman C., Ethier S.P., Welsh J. (1999). SUM-159PT cells: A novel estrogen independent human breast cancer model system. Breast Cancer Res. Treat..

[48] Allan A.L., George R., Vantyghem S.A., Lee M.W., Hodgson N.C., Engel C.J., Holliday R.L., Girvan D.P., Scott L.A., Postenka C.O. (2006). Role of the integrin-binding protein osteopontin in lymphatic metastasis of breast cancer. Am. J. Pathol..

[49] Croker A.K., Allan A.L. (2011). London Regional Cancer Program.

[50] Schulze E.B., Hedley B.D., Goodale D., Postenka C.O., Al-Katib W., Tuck A.B., Chambers A.F., Allan A.L. (2008). The thrombin inhibitor argatroban reduces breast cancer malignancy and metastasis via osteopontin-dependent and osteopontin-independent mechanisms. Breast Cancer Res. Treat..

[51] Furger K.A., Allan A.L., Wilson S.M., Hota C., Vantyghem S.A., Postenka C.O., Al-Katib W., Chambers A.F., Tuck A.B. (2003). β(3) integrin expression increases breast carcinoma cell responsiveness to the malignancy-enhancing effects of osteopontin. Mol. Cancer Res..

[52] Hess D.A., Craft T.P., Wirthlin L., Hohm S., Zhou P., Eades W.C., Creer M.H., Sands M.S., Nolta J.A. (2008). Widespread non-hematopoietic tissue distribution by transplanted human progenitor cells with high aldehyde dehydrogenase activity. Stem Cells.

[53] Hess D.A., Meyerrose T.E., Wirthlin L., Craft T.P., Herrbrich P.E., Creer M.H., Nolta J.A. (2004). Functional characterization of highly purified human hematopoietic repopulating cells isolated according to aldehyde dehydrogenase activity. Blood.

[54] Hess D.A., Wirthlin L., Craft T.P., Herrbrich P.E., Hohm S.A., Lahey R., Eades W.C., Creer M.H., Nolta J.A. (2006). Selection based on CD133 and high aldehyde dehydrogenase activity isolates long-term reconstituting human hematopoietic stem cells. Blood.

[55] Leong H.S., Chambers A.F., Lewis J.D. (2012). Assessing cancer cell migration and metastatic growth in vivo in the chick embryo using fluorescence intravital imaging. Methods Mol. Biol..

[56] Seandel M., Noack-Kunnmann K., Zhu D., Aimes R.T., Quigley J.P. (2001). Growth factor-induced angiogenesis in vivo requires specific cleavage of fibrillar type I collagen. Blood.

