# Supplementary material for: Differential Functional Roles of ALDH1A1 and ALDH1A3 in Mediating Metastatic Behavior and Therapy Resistance of Human Breast Cancer Cells
Source: Int J Mol Sci. 2017 Sep 22;18(10):2039. doi: 10.3390/ijms18102039 (PMC5666721; doi:10.3390/ijms18102039)
Supplement: Supplementary file 1 [file ijms-18-02039-s001.pdf]

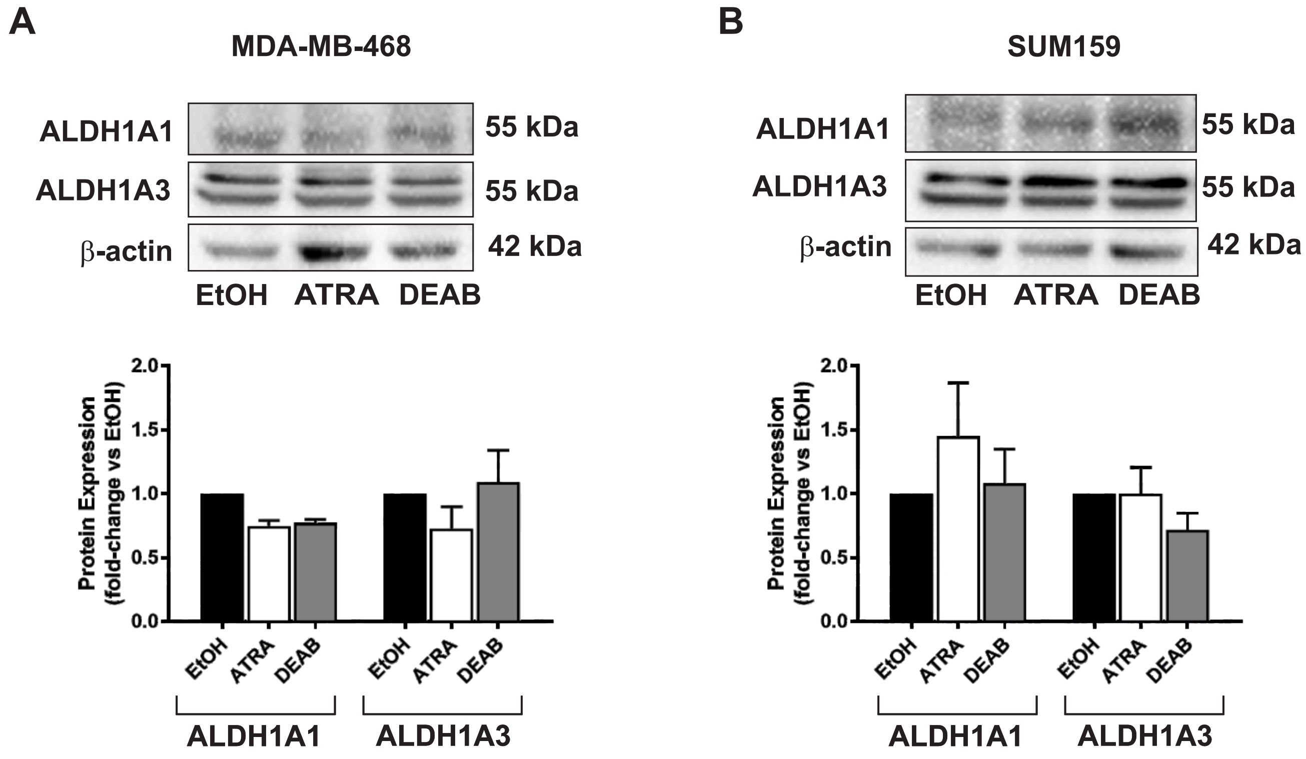

Supplemental Figure S1: Treatment with ATRA or DEAB does not alter protein expression of ALDH1A1 or ALDH1A3 isozymes. ***(A)*** MDA-MB-468 and ***(B)*** SUM159 human breast cancer cells were treated in culture for 48 hours with 5μM ATRA, 100μM DEAB or EtOH as a vehicle control. Cell lysates were collected and subjected to immunoblotting to assess ALDH1A1 and ALDH1A3 protein expression. Top panels show representative immunoblots. Bottom plots show quantitative densitometric analysis (n=3 blots). In all cases, data represents the mean ± SEM normalized to vehicle control.

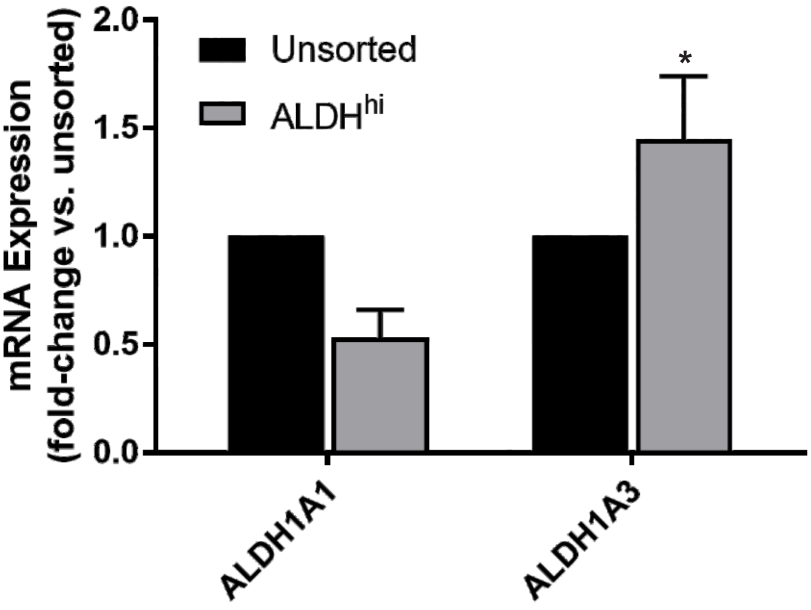

Supplemental Figure S2: ALDH1A3 mRNA expression is higher than ALDH1A1 mRNA expression in sorted ALDH^{hi} breast cancer cell populations. MDA-MB-468 breast cancer cells were subjected to FACS using the Aldefluor® assay as described in the Materials and Methods. Unsorted and sorted ALDH^{hi} cell populations were subjected to qRT-PCR to assess ALDH1A1 and ALDH1A3 expression (n=3). Data represents the mean +/- SEM. * = significantly different than ALDH1A1 ($p<0.05$).
